# Bacteriophages 6phi8, 6phi10, and 6phi13 Isolated from the Therapeutic Cocktail “Sextaphag^®^”

**DOI:** 10.3390/biotech15040072

**Published:** 2026-08-24

**Authors:** Vladislav Kulyabin, Andrey Shadrin

**Affiliations:** Skryabin Institute of Biochemistry and Physiology of Microorganisms, Russian Academy of Sciences, Federal Research Center Pushchino Scientific Center for Biological Research of the Russian Academy of Sciences, Pushchino 142290, Russia

**Keywords:** bacteriophages, genome, *Escherichia* spp., antimicrobial resistance (AMR)

## Abstract

The present study provides the physicochemical and genomic characterization of three *Escherichia* bacteriophages (6phi8, 6phi10, and 6phi13) isolated from the commercial phage preparation “Sextaphag^®^”. For each bacteriophage, lytic activity against *E. coli* MG1655, as well as pH and thermal stability, were determined. Whole-genome sequencing was performed, followed by bioinformatic annotation and comparative genomic analysis. Bacteriophages 6phi8 and 6phi13 belong to the T4-like myoviruses with large genomes (~169 and ~171 kb, respectively), whereas 6phi10 is a T7-like podovirus with a genome size of 40.1 kb. Phage 6phi8 was assigned to the genus *Mosigvirus* of the family *Straboviridae*, 6phi13 was classified within the genus *Tequatrovirus* of the same family, and 6phi10 was identified as a putative novel species of the genus *Berlinvirus* within the family *Autotranscriptaviridae*. The genomes of the studied phages lack genes associated with the lysogenic cycle, as well as virulence and antibiotic resistance determinants. The results expand current knowledge of the genomic properties of phages included in therapeutic cocktails and may contribute to the development of phage preparations against infections caused by *E. coli* and other members of the family *Enterobacteriaceae*.

## 1. Introduction

Antimicrobial resistance (AMR) represents one of the major challenges of modern healthcare. According to a large-scale analysis published by the Global Research on Antimicrobial Resistance (GRAM) Project, more than 1 million people die annually from infections caused by antibiotic-resistant bacteria. Between 1990 and 2021, the cumulative number of such deaths exceeded 36 million. If current trends persist, AMR-associated mortality may reach 1.91 million deaths annually, while the total number of deaths in which resistant bacteria act as a contributing factor may rise to 8.22 million cases per year [1].

The epidemiological situation is further complicated by the rapid spread of antibiotic resistance among Gram-negative bacteria. This is due to the high plasticity of their genomes and the efficient mechanisms of horizontal transfer of resistance determinants, including plasmids, transposons, and integrons [2].

Among clinically significant pathogens, *Escherichia coli* occupies a special place as a facultatively anaerobic Gram-negative bacterium that forms part of the normal human gastrointestinal microbiota. However, it is capable of causing a wide range of infectious diseases upon entering normally sterile compartments of the human body. *E. coli* is responsible for 80–95% of all urinary tract infections and is also one of the leading etiological agents of nosocomial sepsis [3]. Under conditions of a progressively diminishing therapeutic arsenal against AMR pathogens, increasing attention is being directed toward alternative antimicrobial strategies, among which bacteriophage therapy occupies a prominent position. Virulent phages exhibit high specificity toward bacterial hosts while neither affecting eukaryotic cells nor exerting significant impact on the host’s normal microbiota [4].

Nevertheless, the implementation of phage therapy in clinical practice faces several obstacles, including the limited host range of individual bacteriophages, potential immunogenicity upon systemic administration, and the absence of unified regulatory standards [5]. A necessary prerequisite for overcoming these limitations is the detailed characterization of individual phage isolates, both in terms of their biological and physicochemical properties and their genomic organization. This study presents the results of an investigation of *E. coli* bacteriophages isolated from “Sextaphag^®^” (Microgen, Moscow, Russia). “Sextaphag^®^” is the commercial therapeutic bacteriophage cocktail freely available in pharmacies (national drug registration number ЛΠ-№(003826)-(PΓ-RU)). This preparation is a sterile filtrate of phage lysates active against bacteria of the genera *Staphylococcus*, *Streptococcus*, *Proteus* (including *P. vulgaris* and *P. mirabilis*), *Pseudomonas aeruginosa*, *Escherichia coli*, and *Klebsiella pneumoniae* [6]. The complete composition and formulation of the commercial preparation, including the identity and number of individual bacteriophages, as well as detailed information on its validation and clinical use, are not available in publicly accessible sources. Previous studies have characterized individual bacteriophages isolated from Sextaphag^®^. The enterococcal phage iF6 was classified within the genus *Schiekvirus*, while the lytic *E. coli* phage Sxt1 was identified as a member of the genus *Teetrevirus* [7,8].

In the present study, we characterized three additional *Escherichia* bacteriophages, 6phi8, 6phi10, and 6phi13, isolated from the same commercial preparation. For each isolate, lytic activity, pH and thermal stability were evaluated, and whole-genome sequencing was performed, followed by bioinformatic analysis.

## 2. Materials and Methods

### 2.1. Bacterial Strains and Cultivation Conditions

All bacterial strains were part of the laboratory collection. LB (lysogeny broth) medium (10 g/L tryptone, 5 g/L yeast extract, 10 g/L NaCl) and LB agar (1.5% *w*/*v* for the bottom layer and 0.5% *w*/*v* for the top layer) were used for the cultivation of bacteria and bacteriophages. *E. coli* cells were cultured at 37 °C. Bacterial growth was monitored by measuring optical density (OD) at a wavelength of 590 nm using an Expert-003 photometer (Econics-Expert, Moscow, Russia).

### 2.2. Isolation, Purification, and Propagation of Bacteriophages

Phages were isolated from the therapeutic bacteriophage cocktail “Sextaphag^®^” (Microgen, batch P813, December 2016). The double-layer agar method was used for phage isolation and subsequent propagation. *Escherichia coli* MG1655 served as the host strain. A 100 µL volume of the preparation was added to 200 µL of an overnight *E. coli* culture, mixed with soft agar (0.5%), and thoroughly vortexed. The resulting mixture was then poured onto the surface of a solid agar layer (1.5%). Plates were incubated at 37 °C overnight.

Material from the resulting zones of lysis was collected by spot transfer and placed into a separate tube containing 300 µL of SM+ buffer (100 mM NaCl, 1 mM MgSO_4_, 50 mM Tris-HCl pH 7.5, 0.01% gelatin) supplemented with 10 µL of chloroform. The resulting mixture was incubated at 5 °C for 1 h.

Subsequently, a series of dilutions was prepared and used to infect a fresh bacterial culture. For this purpose, 10 µL of the diluted phage samples were added to 200 µL of an overnight *E. coli* culture, mixed with soft LB medium, thoroughly vortexed, and plated onto the surface of a solid agar layer. The plates were incubated at 37 °C overnight. The described procedure of sequential passaging was repeated four times.

To obtain a high-titer phage preparation, 500 µL of phage suspension was added to 250 mL of an *E. coli* MG1655 culture (optical density OD_590_ ≈ 0.5). Incubation was carried out at 37 °C with orbital shaking (200 rpm) until a marked decrease in optical density was observed, indicating bacterial cell lysis. After lysis of the bacterial culture, NaCl (14 g) and chloroform (2.5 mL) were added to the flask, and incubation was continued for 30 min.

Cellular debris was removed by centrifugation at 16,000× *g* for 15 min at 7 °C. Viral particles were precipitated using polyethylene glycol (PEG-8000) to a final concentration of 10% (*w*/*v*), followed by incubation at 4 °C for 2 h and a second centrifugation step at 16,000× *g* for 15 min. The resulting pellet was resuspended in SM+ buffer and subsequently subjected to filtration through a 0.22 µm pore-size membrane. The concentrated phage preparation was stored at 4 °C.

### 2.3. Killing Assay

Bacterial growth was monitored by measuring optical density at 595 nm using a FilterMax F5 plate reader (Molecular Devices, San Jose, CA, USA). To prepare the inoculum, 1 mL of an overnight *E. coli* culture was transferred into a flask containing liquid LB medium and incubated until an optical density of OD_595_ ≈ 0.2 was reached, corresponding to a cell concentration of approximately 5 × 10^8^ CFU/mL. The bacterial suspension was then distributed into the wells of a 48-well plate, after which the phage preparation was added to achieve different multiplicities of infection (MOI): 0.01, 0.1, 1, and 10. Control samples consisted of *Escherichia coli* MG1655 cultures supplemented with SM+ buffer without phage particles. LB medium supplemented with SM+ buffer was used as a blank control.

Cells were cultured at 37 °C for 4 h, with optical density (595 nm) measured every 5 min. All experiments were performed in three independent biological replicates. Analysis and visualization of the resulting growth curves were carried out using GraphPad Prism software (v10.6.1).

### 2.4. pH and Thermal Stability of Bacteriophages

Thermal stability of bacteriophages was assessed by incubating 300 µL aliquots of phage suspension (titer 5 × 10^8^ PFU/mL) across a temperature range of 10–80 °C for 1 h. After thermal treatment, samples were rapidly cooled on ice for 10 min, followed by serial dilution and determination of viable virions using the double-layer agar method.

To investigate the effect of acidic and alkaline conditions, phage suspensions were added to buffer solutions with different pH values (2.2–12), adjusting the final concentration of viral particles to 5 × 10^8^ PFU/mL. The following buffer systems (final concentration 50 mM) were used: glycine–HCl (pH 2.2 and 3.0), sodium acetate (pH 4.0 and 5.0), phosphate buffer (pH 6.0–8.0), glycine–NaOH (pH 9.0 and 10.0), and Na_2_HPO_4_–NaOH (pH 11.0 and 12.0). Samples were incubated at 25 °C for 1 h, after which serial dilutions were prepared, and phage titers were determined using the double-layer agar method.

All experiments were performed in three independent biological replicates. Data processing and graphical representation were carried out using GraphPad Prism software (v10.6.1).

### 2.5. Extraction of Phage DNA, Sequencing, and Genome Assembly

Phage preparations obtained at the previous step were treated with DNase I (Dia-M, 3911.2000) and RNase A (Thermo Scientific, Vilnius, Lithuania, EN0531) at 37 °C for 1 h with intermittent mixing to remove free nucleic acids. Capsid disruption was performed using proteinase K and sodium dodecyl sulfate (SDS). Phage DNA was then extracted using the standard phenol–chloroform method. Further purification of the obtained DNA was carried out using a Magen purification kit (Guangzhou Magen Biotechnology Co., Guangzhou, China, D311102).

The quality of the extracted DNA was assessed by electrophoresis in a 1% agarose gel, while DNA concentration was measured using a NanoPhotometer Pearl P-360 spectrophotometer (Implen GmbH, Munich, Germany).

The preparation of paired-end DNA libraries was performed using the VAHTS Universal Plus DNA Library Prep Kit for Illumina V2 (Vazyme, Nanjing, China). Sequencing was carried out on a SURFSeq 5000 platform (GeneMind, Shenzhen, China). Library preparation and bacteriophage genome sequencing were performed by Sesana LLC (Moscow, Russia) at the Genomed laboratory facility.

Raw sequencing data were subjected to primary processing, including subsampling of reads, removal of adapter sequences, and quality filtering using Trimmomatic (v0.40) and fastp (v1.1.0).

Genome assembly was performed using the SPAdes software package (v3.15.5) [9], which enabled the generation of contiguous sequences. Subsequent assembly polishing was carried out using Pilon (v1.24) in three successive rounds. Visual assessment of assembly quality was performed using Bandage, while coverage distribution was analyzed in Tablet. Prediction of open reading frames and genome annotation were conducted using the Pharokka pipeline (database v1.2.0; Prodigal algorithm) [10]. Identification of tRNA genes was carried out using tRNAscan-SE (v2.0.12) and ARAGORN (v1.2.41). Circular genome maps were visualized using the Proksee platform [11].

The assembled genomes were deposited in the GenBank database under accession numbers PZ311405 (vB_Eco_6phi10), PZ311406 (vB_Eco_6phi8), and PZ311407 (vB_Eco_6phi13). Hereafter, the bacteriophages are referred to as 6phi10, 6phi8, and 6phi13, respectively.

### 2.6. Comparative Genomics

To identify related bacteriophages, searches for similar genomes were performed using BLASTn(v2.17.0) and BLASTx (v2.17.0), with the complete genome sequence used as the query. Additionally, genomes of closely related phages selected based on BLAST results were included in the comparative analysis.

Predicted proteomes derived from the annotated genomes were compared with proteomes of known viruses using the ViPTree software package (v4.0), enabling assessment of the phylogenetic and taxonomic positions of the studied bacteriophages [12]. For nucleotide-level analysis, the Genome-BLAST Distance Phylogeny (GBDP) method implemented in the VICTOR platform was applied [13]. Visualization of the resulting proteome-based phylogenetic trees was performed using the iTOL platform (v7.5.1) [14].

Multiple sequence alignment of protein amino acid sequences was performed using Clustal Omega (v1.2.4) on the EMBL-EBI platform.

### 2.7. Restriction Analysis

Phage 6phi10 genomic DNA (1 μg) was digested with XbaI and NdeI restriction enzymes (New England Biolabs, Ipswich, MA, USA) in a total reaction volume of 50 μL using 1× CutSmart Buffer for 2 h at 37 °C. The resulting DNA fragments were separated by electrophoresis in a 15 cm 1% (*w*/*v*) agarose gel prepared in 1× TAE buffer (40 mM Tris, 20 mM acetic acid, and 1 mM EDTA) at 150 V for 1.5 h. A DNA molecular size marker (M29 + M33, SibEnzyme, Novosibirsk, Russia) was used for fragment-size estimation. DNA fragments were visualized by ethidium bromide staining under UV illumination. The in silico restriction profile was generated using SnapGene v. 3.2.1 (GSL Biotech LLC, Chicago, IL, USA) based on the assembled genome sequence of phage 6phi10 and the same restriction enzymes.

Restriction fragments observed after electrophoresis were assigned to the predicted in silico fragments according to their estimated sizes. Terminal fragments were identified as those predicted to span the left and right genome termini and the corresponding DTR regions.

## 3. Results

### 3.1. Isolation of Bacteriophages

**Figure 1:**
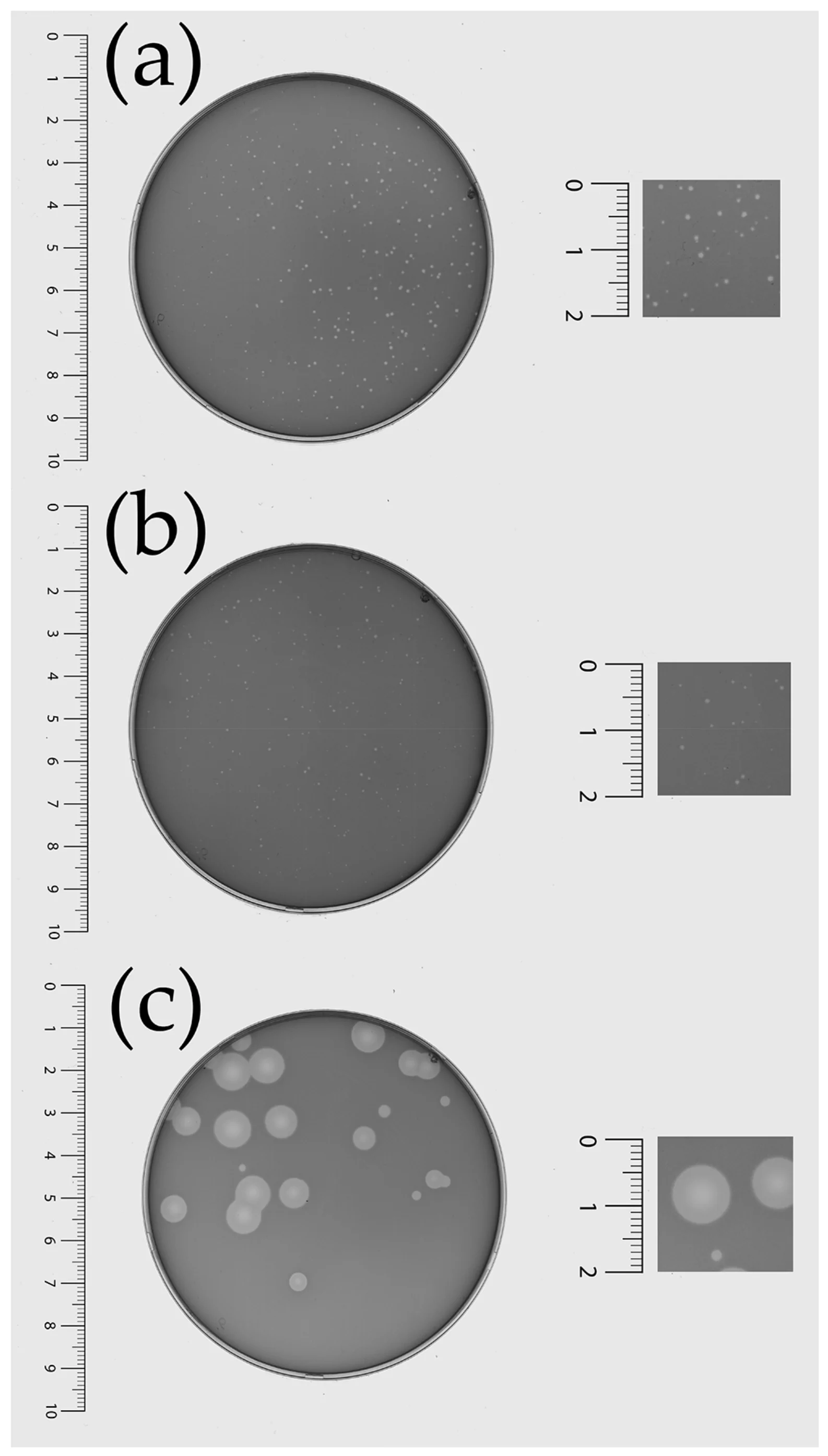
Plaque morphology of bacteriophages 6phi8 (**a**), 6phi13 (**b**), and 6phi10 (**c**) on an *E. coli* MG1655 lawn.

*Escherichia* bacteriophages 6phi8, 6phi10, and 6phi13 were isolated from the commercial phage cocktail “Sextaphag^®^”. Bacteriophages 6phi8 and 6phi13 formed clear plaques up to 1 mm in diameter on an *E. coli* MG1655 lawn (Figure 1a,b). Phage 6phi10 formed clear plaques up to 9 mm in diameter on an *E. coli* MG1655 lawn (Figure 1c).

### 3.2. Thermal and pH Stability

Analysis of the pH and thermal stability of bacteriophages allows determination of optimal storage conditions both during transportation and throughout the successive stages of experimental use.

**Figure 2:**
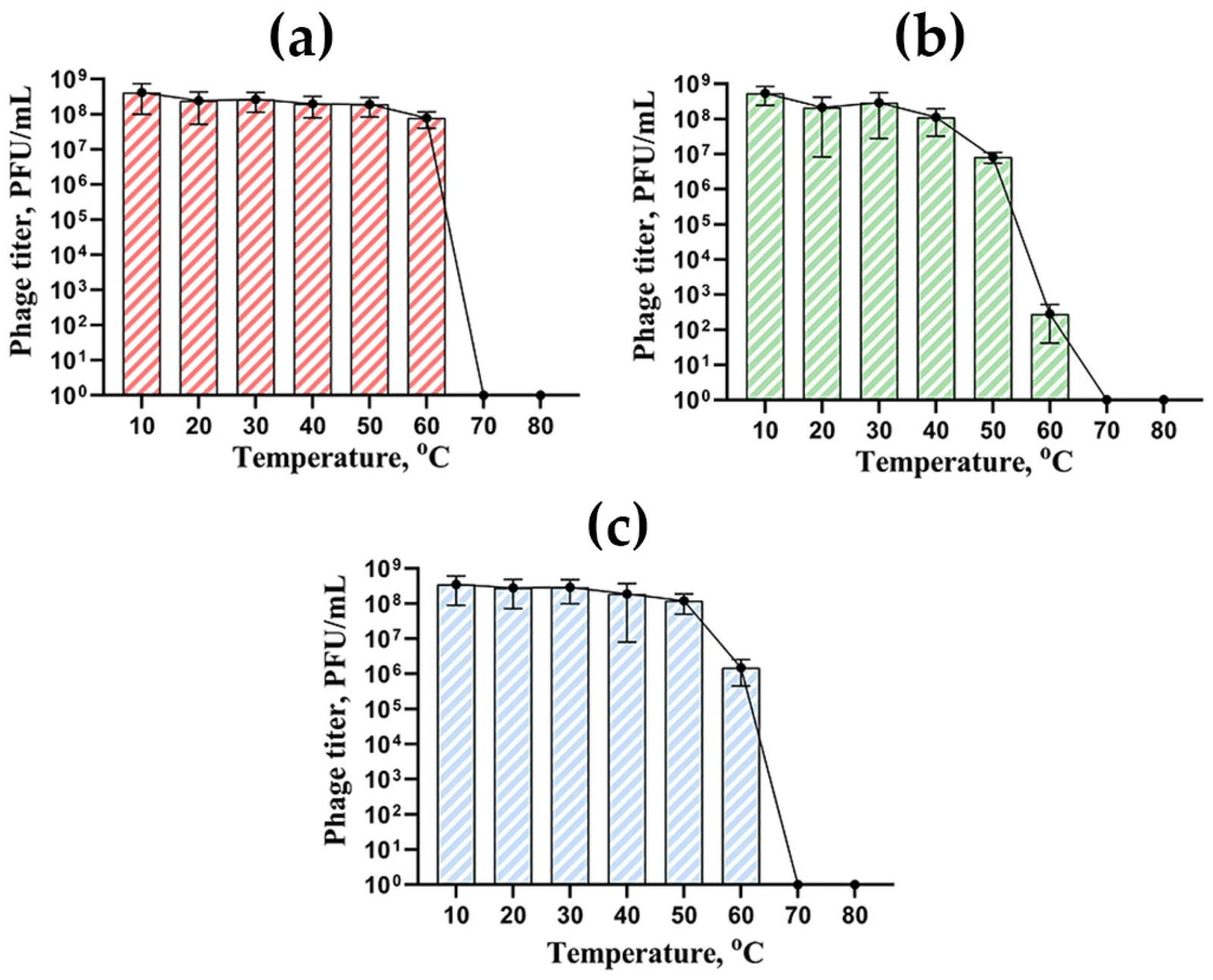
Thermal stability of bacteriophages 6phi8 (**a**), 6phi10 (**b**), and 6phi13 (**c**). Phage titers after 1 h of incubation at temperatures ranging from 10 to 80 °C.

Bacteriophage 6phi8 was characterized by a broad range of thermal stability and retained its initial titer after 1 h of incubation at temperatures ranging from 10 to 60 °C (Figure 2a). For bacteriophage 6phi10, a decrease in titer was observed already at 50 °C (approximately one order of magnitude), whereas at 60 °C a more pronounced reduction was detected (up to six orders of magnitude) (Figure 2b). Bacteriophage 6phi13 demonstrated thermal stability within the temperature range of 10–50 °C; however, at 60 °C its titer decreased by approximately two orders of magnitude (Figure 2c). None of the studied phages retained viability after incubation at 70 or 80 °C.

**Figure 3:**
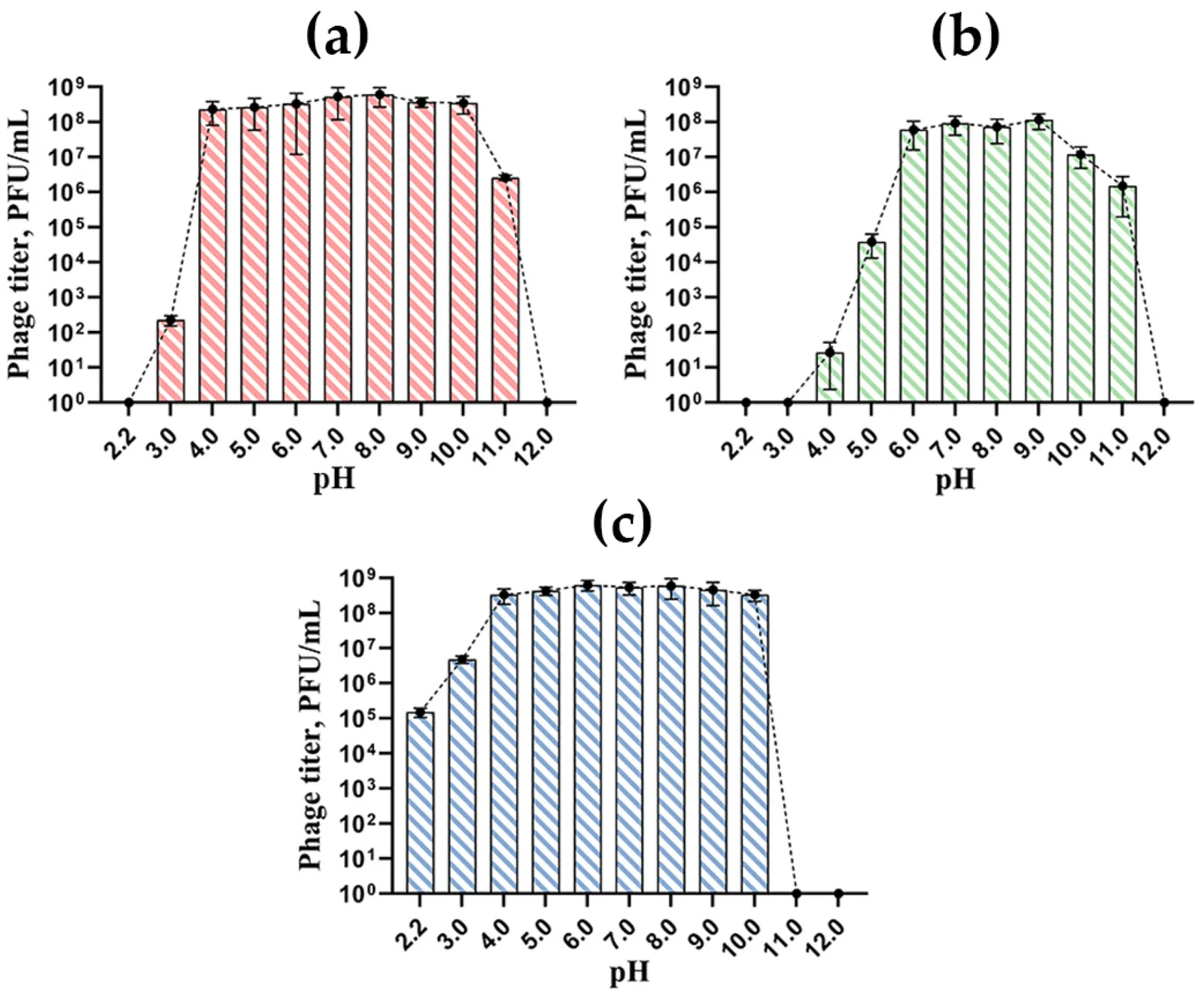
pH stability of bacteriophages 6phi8 (**a**), 6phi10 (**b**), and 6phi13 (**c**). Phage titers after 1 h of incubation at pH values ranging from 2.2 to 12.0.

Analysis of stability under different pH conditions showed that bacteriophage 6phi8 possesses a broad pH stability range and retains its titer after 1 h of incubation at pH values from 4.0 to 10.0 (Figure 3a). Bacteriophage 6phi10 exhibited a narrower stability range, remaining stable within the pH interval of 6.0–9.0 without significant reduction in titer (Figure 3b). Phage 6phi13 demonstrated pH stability similar to that of phage 6phi8, maintaining viability in the pH range from 4.0 to 10.0 (Figure 3c). At pH 2.2, bacteriophage 6phi8 showed an approximately three-orders-of-magnitude reduction in titer, whereas the other studied phages were completely inactivated under these conditions.

### 3.3. Killing Assay

**Figure 4:**
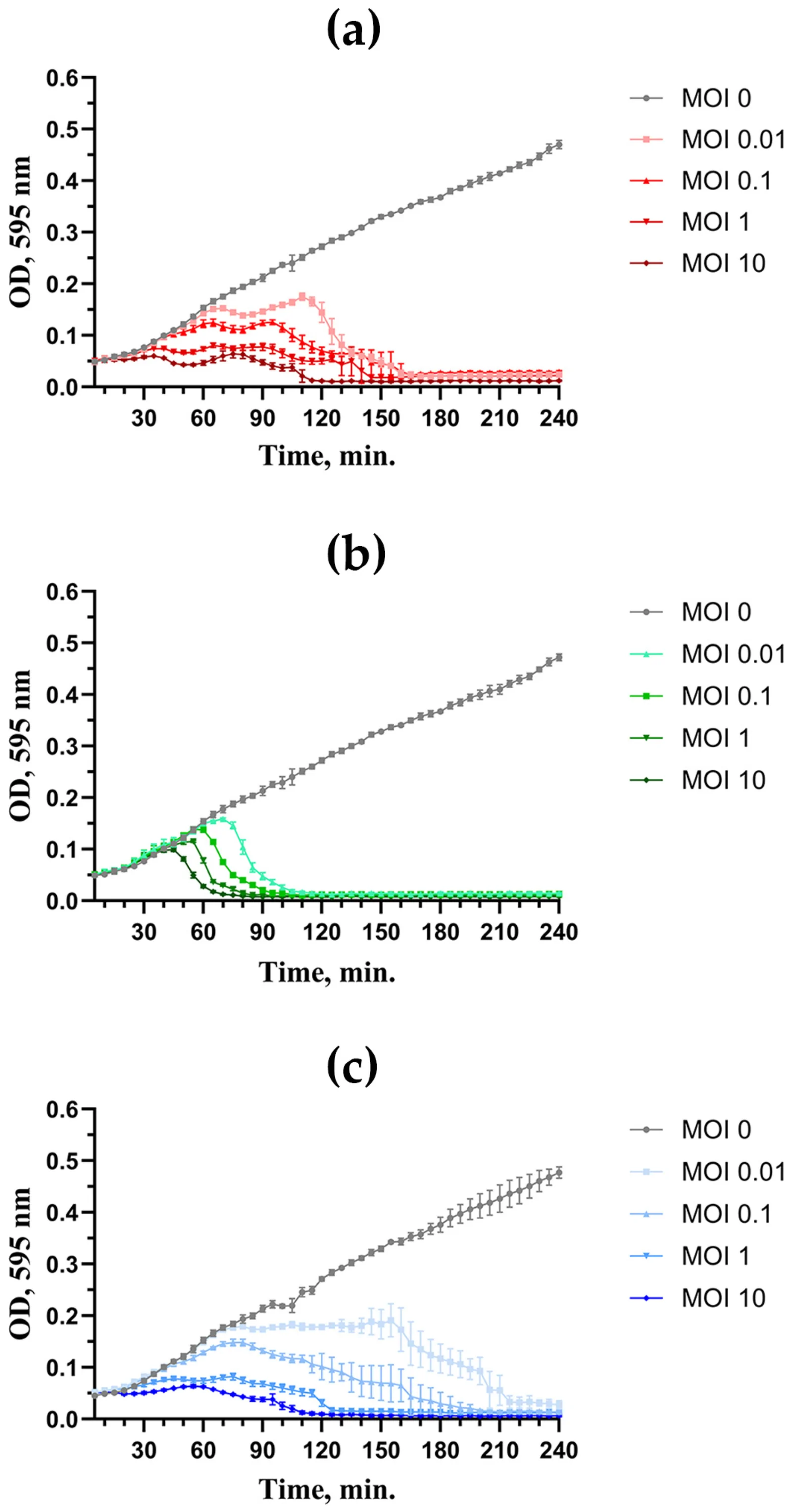
Growth curves of *E. coli* MG1655 following infection with bacteriophages 6phi8 (**a**), 6phi10 (**b**), and 6phi13 (**c**) at different multiplicities of infection (MOI).

The lytic activity of *Escherichia* bacteriophages 6phi8, 6phi10, and 6phi13 against *Escherichia coli* MG1655 was evaluated at different multiplicities of infection (MOI: 0.01, 0.1, 1, and 10) by comparing the growth dynamics of infected and control (uninfected) cultures. The experiment was performed using a bacterial culture in the exponential growth phase (OD_595_ ≈ 0.2). The results demonstrated a pronounced dependence of the inhibitory effect on the initial phage concentration: as the MOI increased, suppression of bacterial population growth as well as lysis of the bacterial culture were observed (Figure 4).

### 3.4. Genomic Organization of Bacteriophages 6phi8, 6phi10, and 6phi13

**Figure 5:**
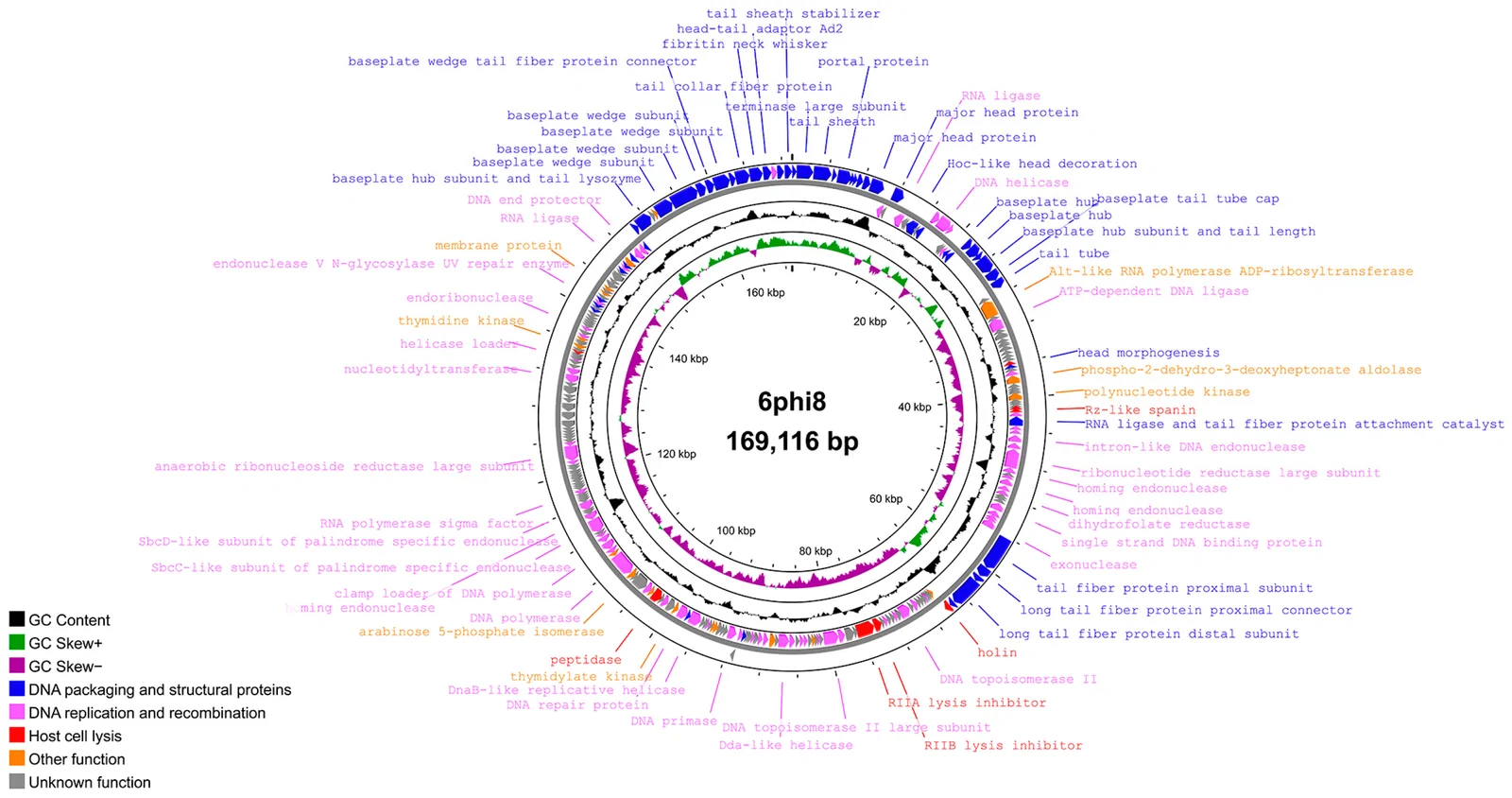
Genome map of *Escherichia* bacteriophage 6phi8. Functionally annotated open reading frames (ORFs) are grouped according to their predicted functions.

The genome of bacteriophage 6phi8 consists of double-stranded DNA with a length of 169,116 bp and a GC content of 37.45%. A total of 261 CDSs were identified in the genome. The coding density is 94.83%, indicating a highly compact genome organization (Figure 5). No tRNA genes were detected in the analyzed genome. Genes associated with virion morphogenesis and genome packaging are predominantly localized on one DNA strand, whereas genes involved in replication and other metabolic processes are distributed on the opposite strand. The genome of 6phi8 exhibits a modular organization. Distinct gene clusters associated with genome packaging and virion morphogenesis were identified, including genes encoding the small and large subunits of terminase, portal protein, capsid proteins, tail sheath, tail tube, baseplate, and tail fibers. In addition, the genome contains genes presumably involved in nucleic acid replication and metabolism, including DNA polymerase, primase, helicase, exonuclease, ligase, topoisomerase, and ribonucleotide reductase. The lysis module includes holin, peptidase, and Rz-like spanin proteins. Ten genes encoding putative homing endonucleases were also identified. Genes associated with the lysogenic cycle, as well as virulence or antibiotic resistance determinants, were not detected within the scope of the annotation.

**Figure 6:**
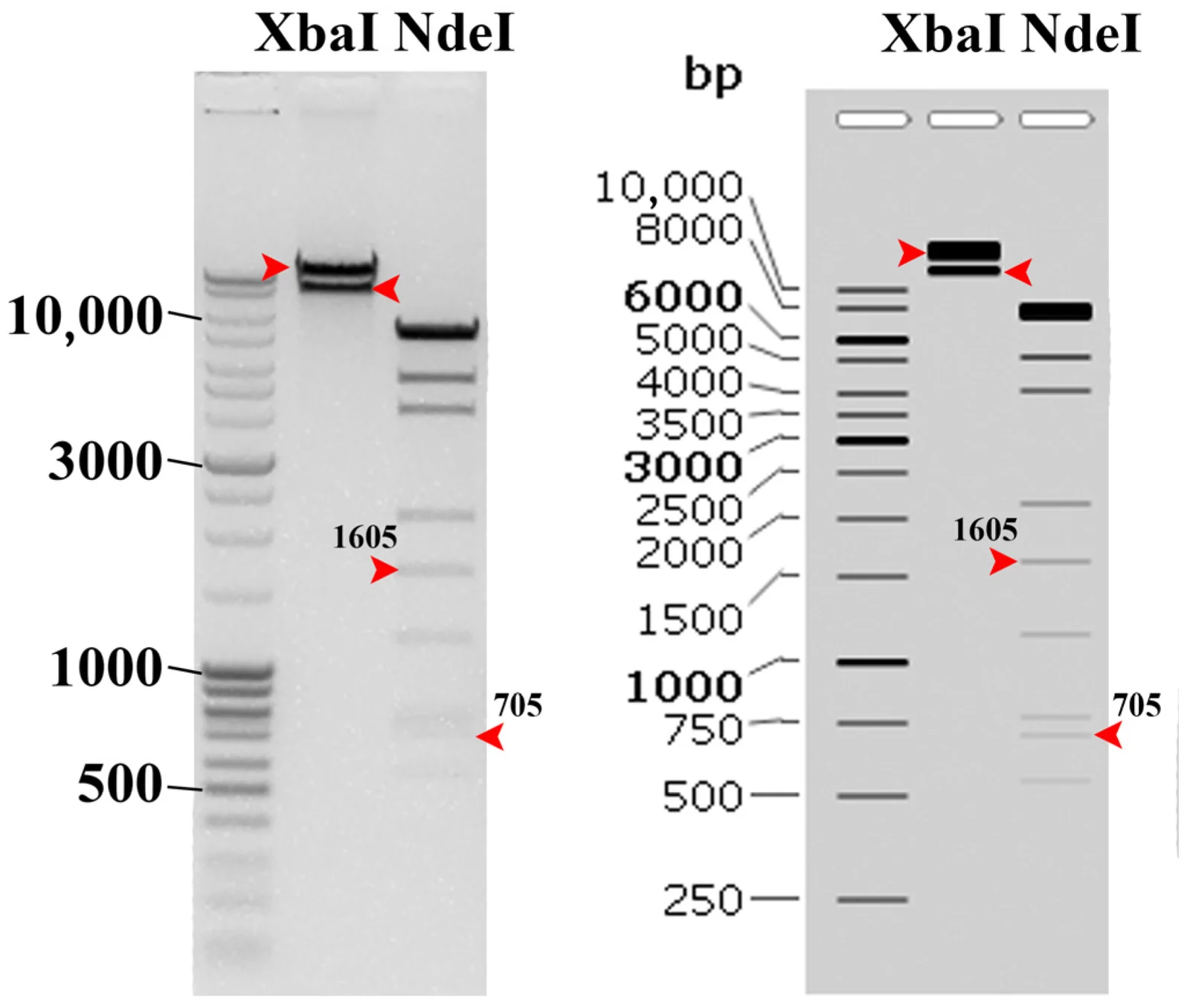
Restriction profile of phage 6phi10 DNA in vitro (**left panel**) and in silico (**right panel**). Terminal fragments containing DTRs are indicated by red triangles.

The genome of bacteriophage 6phi10 consists of double-stranded DNA with a length of 40,100 bp and a GC content of 48.75%. A total of 44 CDSs were identified, with a coding density of 89.4%. No tRNA genes were detected in the analyzed genome. Identification of direct terminal repeats (DTRs) was performed through comparison with the genomes of closely related bacteriophages in which these elements had previously been described. Restriction endonuclease digestion of purified 6phi10 genomic DNA, followed by agarose gel electrophoresis, produced terminal fragments (Figure 6, red triangles) whose sizes matched those predicted by in silico digestion of the assembled genome. This agreement between the experimental and predicted restriction profiles provides independent support for the proposed genome termini and DTR boundaries, complementing the coverage-based evidence described above. Putative direct terminal repeats of 195 bp were found to exhibit sequence conservation and were further supported by a characteristic read coverage profile at the genome termini, as well as by the results of restriction analysis of genomic DNA.

**Figure 7:**
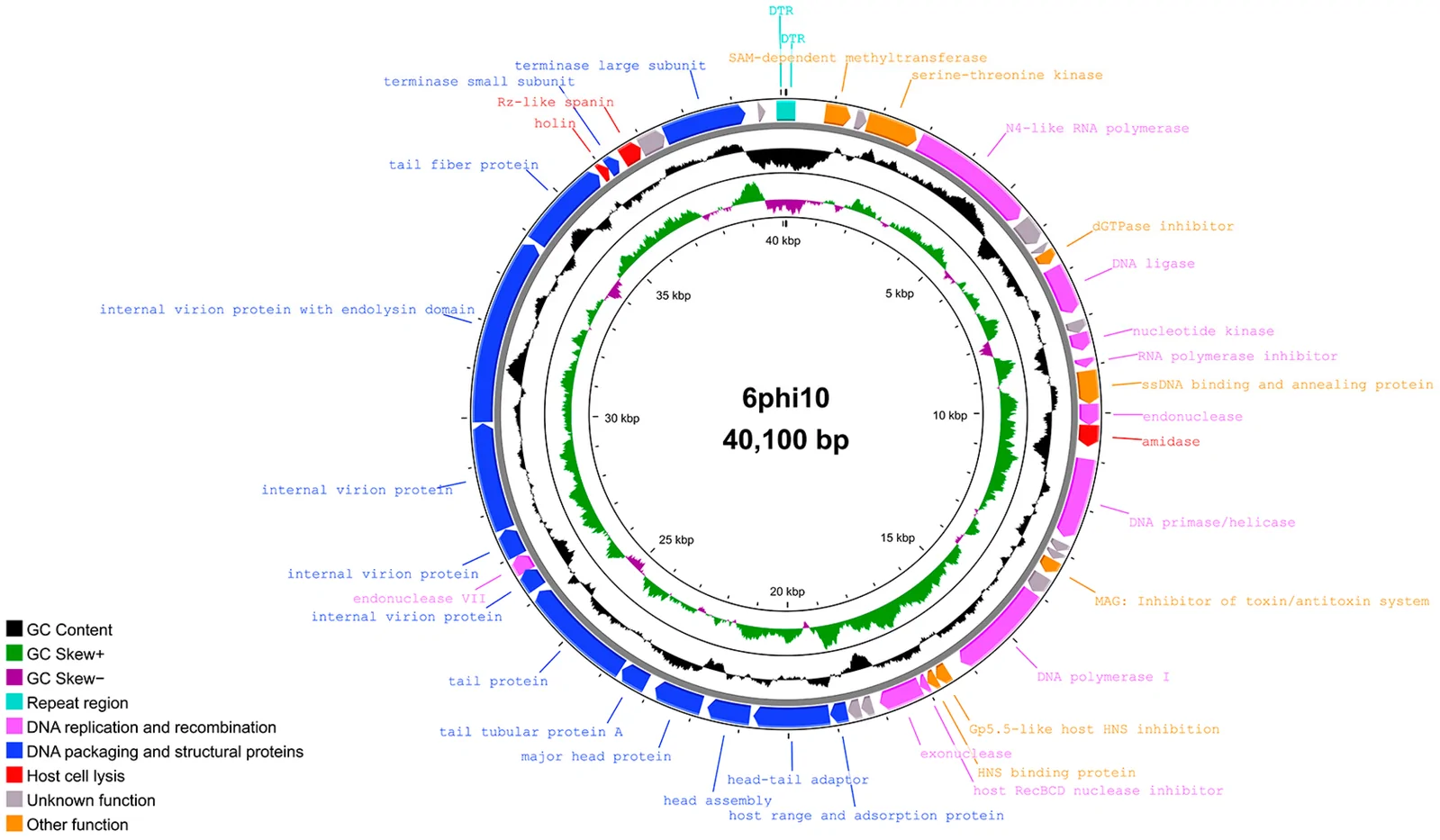
Genome map of *Escherichia* bacteriophage 6phi10. Functionally annotated open reading frames (ORFs) are grouped according to their predicted functions.

All identified CDSs are localized on a single DNA strand. The genome of 6phi10 exhibits a modular organization (Figure 7). It contains genes associated with replication and nucleic acid metabolism, including DNA polymerase I, DNA primase/helicase, DNA ligase, exonuclease, and single-stranded DNA-binding protein (SSB). The phage genome also harbors genes whose products are involved in transcriptional regulation and interactions with host–cell proteins, including an N4-like RNA polymerase, inhibitors of bacterial RNA polymerase, as well as H-NS and RecBCD inhibitors. The structural module includes genes encoding the head–tail adaptor, head assembly protein, major capsid protein, tail proteins, tail fiber protein, and internal virion proteins. The genome also contains genes associated with host–cell lysis, including amidase, holin, and Rz-like spanins, as well as genes encoding the small and large terminase subunits. No integrases, lysogeny-associated markers, antibiotic resistance genes, or virulence determinants were identified.

**Figure 8:**
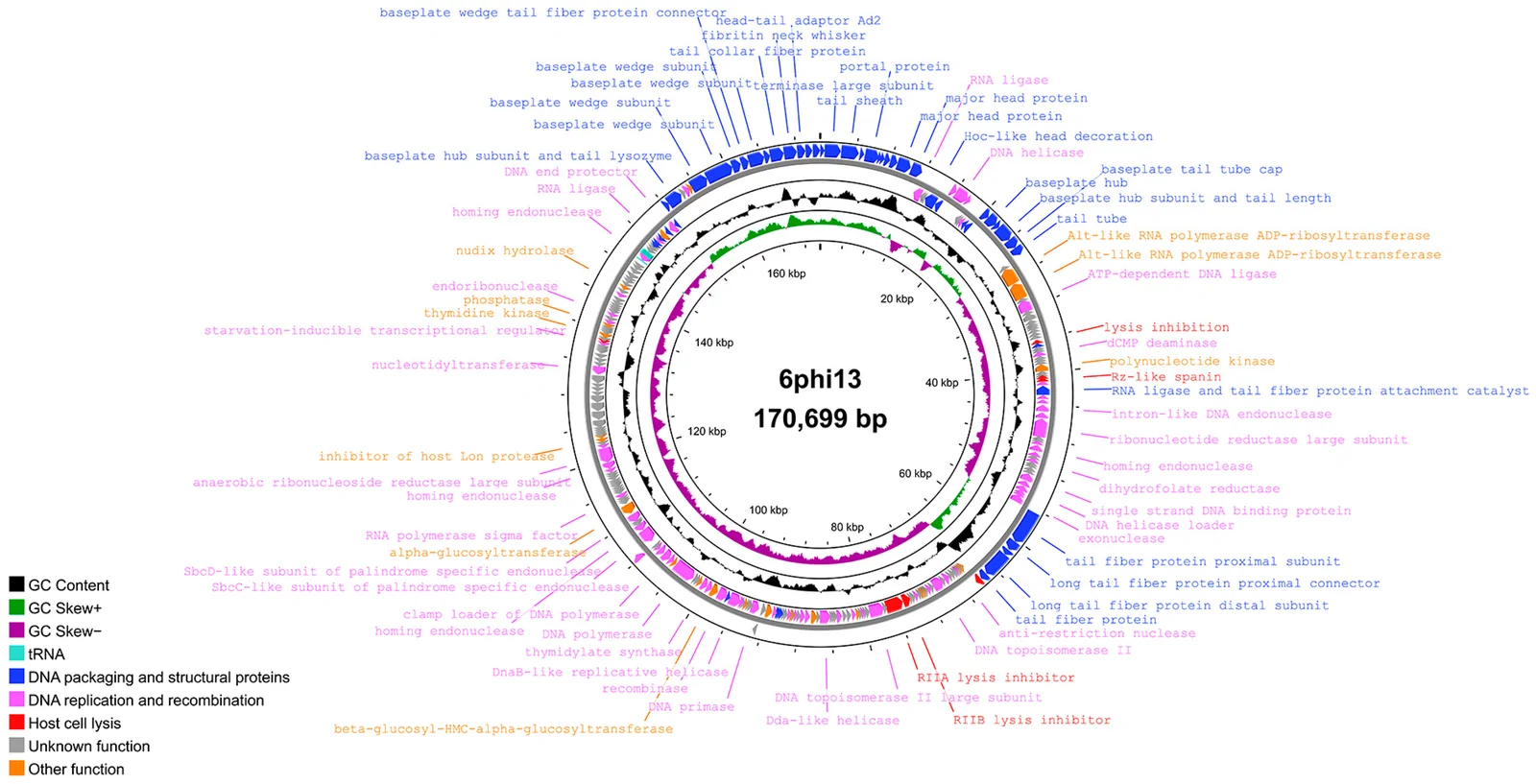
Genome map of *Escherichia* bacteriophage 6phi13. Functionally annotated open reading frames (ORFs) are grouped according to their predicted functions.

The genome of bacteriophage 6phi13 consists of double-stranded DNA with a length of 170,699 bp and a GC content of 35.28%. A total of 270 open reading frames were predicted in the genome, with coding sequences accounting for 94%, reflecting a high degree of genome compaction (Figure 8). Eight tRNA genes were identified in the genome. Phage 6phi13 is characterized by an uneven distribution of genes between the DNA strands, with the majority of CDSs located on one strand. Genes involved in virion morphogenesis and genome packaging are located on the same strand, whereas genes associated with replication and nucleic acid metabolism are located on the opposite strand. The genome of 6phi13 exhibits a pronounced modular architecture. It contains gene clusters associated with virion formation and DNA packaging, including components of the capsid and tail apparatus, as well as terminase subunits and the portal protein. In addition, it harbors genes involved in replication and cellular metabolic processes. Genes responsible for host cell lysis (holin, amidase, and Rz-like spanins) are also present. Furthermore, nine genes encoding homing endonucleases were identified. According to the annotation results, no genes associated with the lysogenic cycle, virulence determinants, or antibiotic resistance were detected.

The genomes of all the studied bacteriophages are characterized by a pronounced modular architecture, while differing in size, composition, and repertoire of functional elements. A summary of the genomic characteristics of phages 6phi8, 6phi10, and 6phi13 is presented in Table 1.

### 3.5. Comparative Genomics

Homology searches were performed using BLASTn against the NCBI nr database, with the genome of bacteriophage 6phi8 used as the query. As a result, a set of closely related phages was identified with the following nucleotide identity values: NRG-P0074 (PQ480333.1, 96.47%), a20 (OQ067374.1, 93.9%), JM17 (PP400779.1, 89.76%), vB_EcoM_WFK (MK373775.1, 85.72%), vB_EcoM_WFL6982 (MK047718.1, 85.71%), p000y (OY726582.1, 90.84%), vB_Eco_NicPhage (OZ035759.1, 90.64%), mar005p1 (OR352941.1, 89.65%), and Shigella phage JK45 (NC_054940.1, 91.09%).

**Figure 9:**
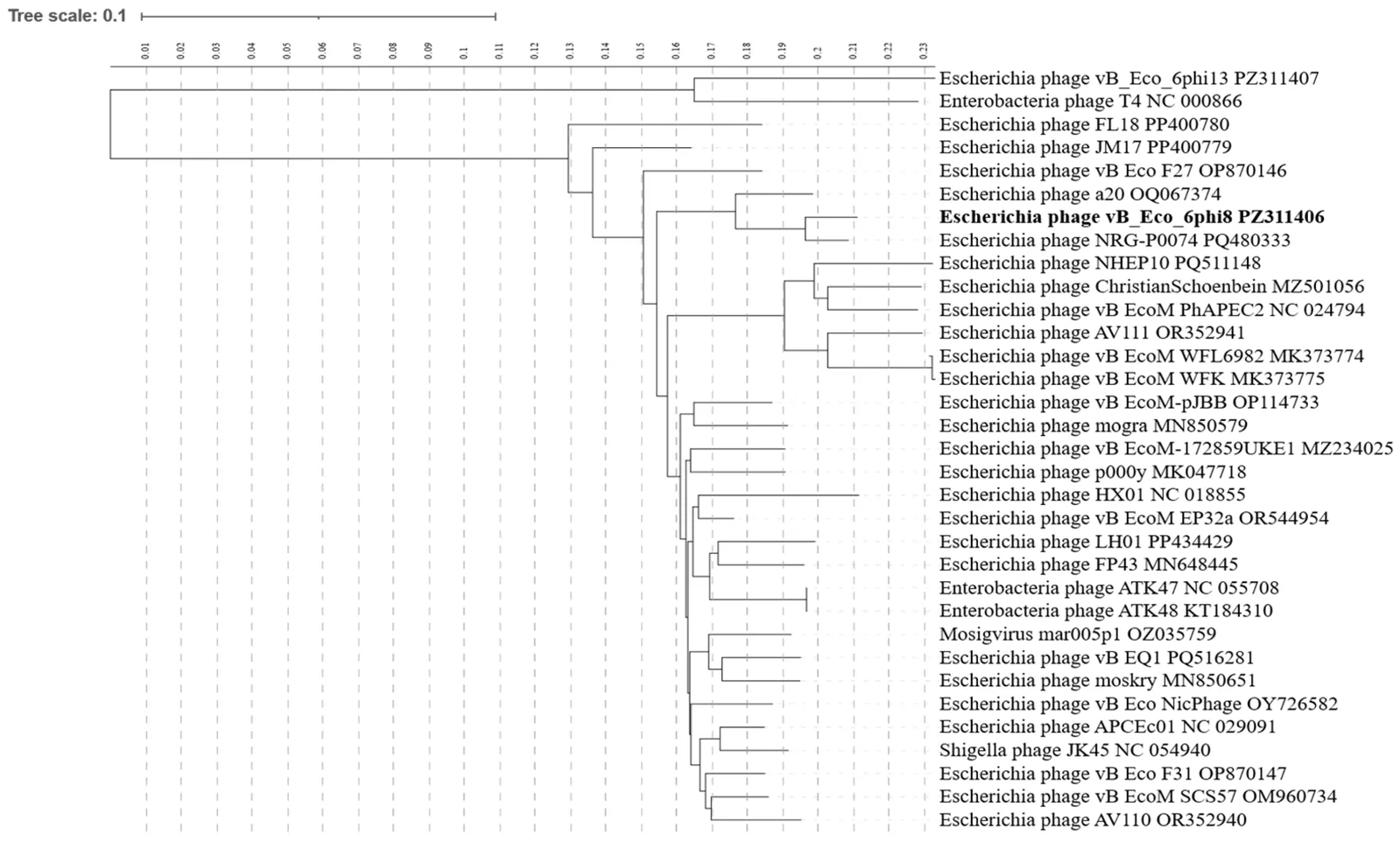
Phylogenetic tree of bacteriophage vB_Eco_6phi8 constructed based on comparative analysis of protein sequences.

Complete genomes of the 30 most closely related phages were retrieved from the GenBank database and used for comparative proteome analysis based on ORFs, employing ViPTree (v4.0) and VICTOR software (formula D6). The resulting phylogenetic tree reflects the evolutionary relationships between phage 6phi8 and its closest relatives (Figure 9).

Analysis using VICTOR (D6) showed that bacteriophage 6phi8 forms a distinct clade with phages NRG-P0074 (PQ480333.1) and a20 (OQ067374.1), which belong to the genus *Mosigvirus*. The nucleotide identity level between phage 6phi8 and the annotated phages NRG-P0074 and a20 exceeds the ICTV species demarcation threshold (95% or more to classify phages into the same species) [15]. Therefore, all three phages (6phi8, NRG-P0074, and a20) can be considered representatives of different strains within a single species belonging to the genus *Mosigvirus*, subfamily *Tevenvirinae*, family *Straboviridae*, order *Pantevenvirales*, class *Caudoviricetes*, phylum *Uroviricota*.

**Figure 10:**
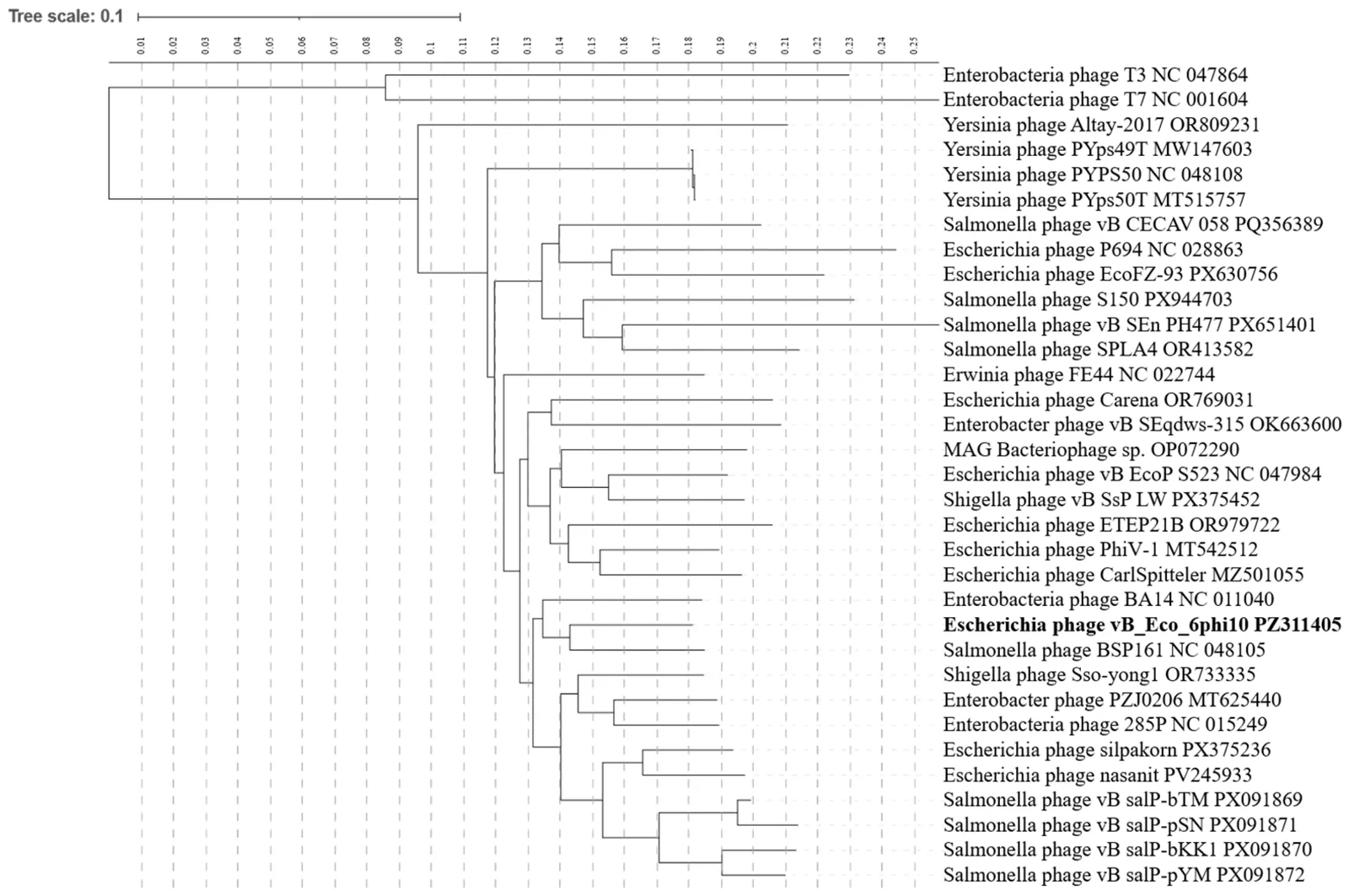
Phylogenetic tree of bacteriophage vB_Eco_6phi10 constructed based on comparative analysis of protein sequences.

Similar genomes identified for bacteriophage 6phi10 in BLASTn included *Erwinia* phage FE44 (NC_022744.1, 88.44%), *Salmonella* phage S150 (PX944703.1, 81.92%) and phage BSP161 (NC_048105.1, 91.25%), *Shigella* phage vB_SsP_LW (PX375452.1, 88.65%), *Enterobacter* phages vB_SEqdws-315 (OK663600.1, 85.77%) and 285P (NC_015249.1, 86.79%), *Escherichia* phage ETEP21B (OR979722.1, 85.7%), as well as *Yersinia* phages PYps50T (MT515757.1, 88.19%), PYps49T (MW147603.1, 88.19%), and PYPS50 (NC_048108.1, 88.16%). For comparative analysis, genomes of the 30 closest phages were retrieved from the GenBank database. The phylogenetic tree constructed using ViPTree illustrates the evolutionary relationships of phage 6phi10 (Figure 10).

Pairwise nucleotide identity between the genome of bacteriophage 6phi10 and phages FE44, BSP161, and BA14 was 88.44%, 91.25%, and 87.94%, respectively. According to the current ICTV demarcation criteria (≥70% and ≥95% nucleotide genome identity for genus- and species-level classification, respectively) [15], bacteriophage 6phi10 should be classified as a new species within the genus *Berlinvirus* (subfamily *Studiervirinae*, family *Autotranscriptaviridae*, order *Autographivirales*, class *Caudoviricetes*, phylum *Uroviricota*).

**Figure 11:**
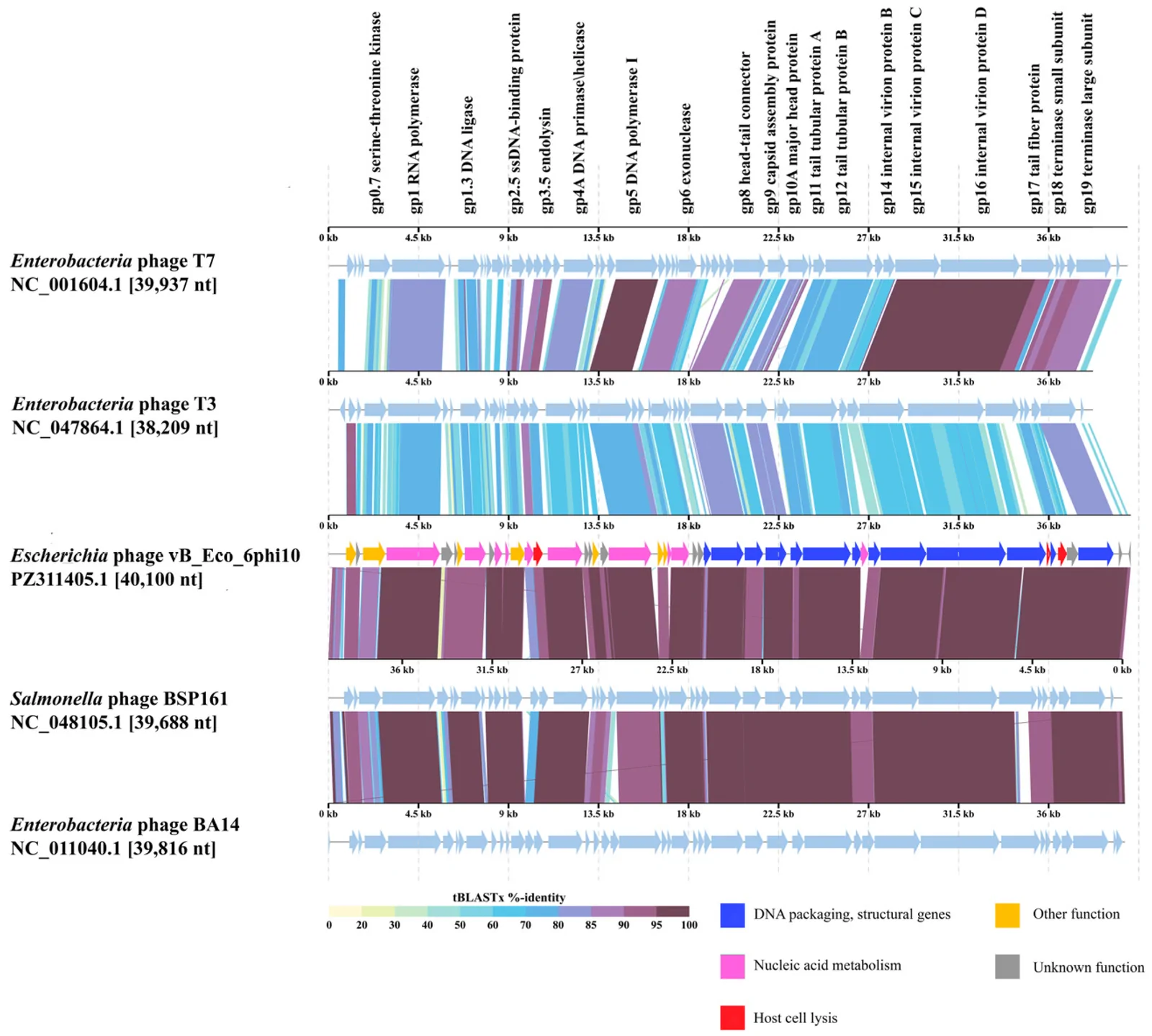
Pairwise comparison of the complete genomes of bacteriophage vB_Eco_6phi10 and related phages performed using tBLASTx.

Figure 11 presents a linear genome comparison diagram obtained using tBLASTx and illustrating pairwise similarity between phage 6phi10 and its closest related phages, *Salmonella* phage BSP161 and *Enterobacteria* phage BA14, as well as phages T3 and T7.

Homologous genomic sequences identified for bacteriophage 6phi13 using BLASTn included *Shigella* phages SHFML-11 (NC_030953.1, 95.06%), SHFML-26 (NC_031011.1, 93.39%), vB_Shso_BCPShso_001 (PV295201.1, 93.09%), and JK23 (MK962752.1, 91.99%), as well as *Escherichia* phages vB_Eco_BCPEco_001 (PV295200.1, 92.99%), UTI-E2 (OL870316.1, 90.15%), vB_EcoM_G4507 (NC_054919.1, 88.97%), vB_EcoM_ESCO58 (OM386666.1, 92.31%), and Adolf Portmann (MZ501046.1, 87.55%).

**Figure 12:**
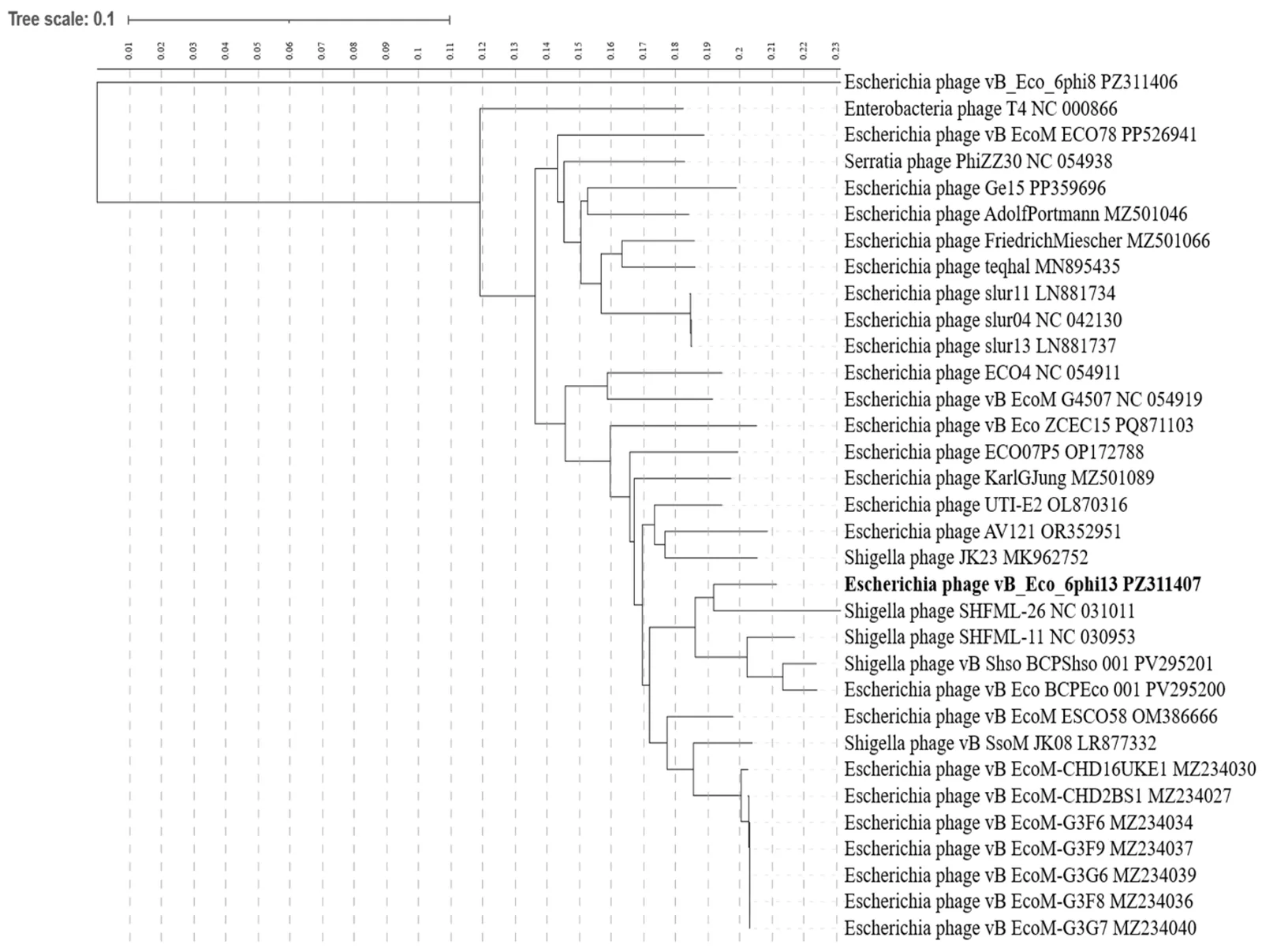
Phylogenetic tree of bacteriophage vB_Eco_6phi13 constructed based on comparative analysis of protein sequences.

To analyze the evolutionary relationships of phage 6phi13, a phylogenetic tree was constructed using ViPTree based on 30 closely related genomes selected from the GenBank database (Figure 12). Phage 6phi13 exhibits more than 95% nucleotide identity with *Shigella* phage SHFML-11, which, according to ICTV criteria, indicates that they belong to the same species. Thus, bacteriophage 6phi13 is classified within the genus *Tequatrovirus*, subfamily *Tevenvirinae*, family *Straboviridae*, order *Pantevenvirales*, class *Caudoviricetes*, phylum *Uroviricota*.

**Figure 13:**
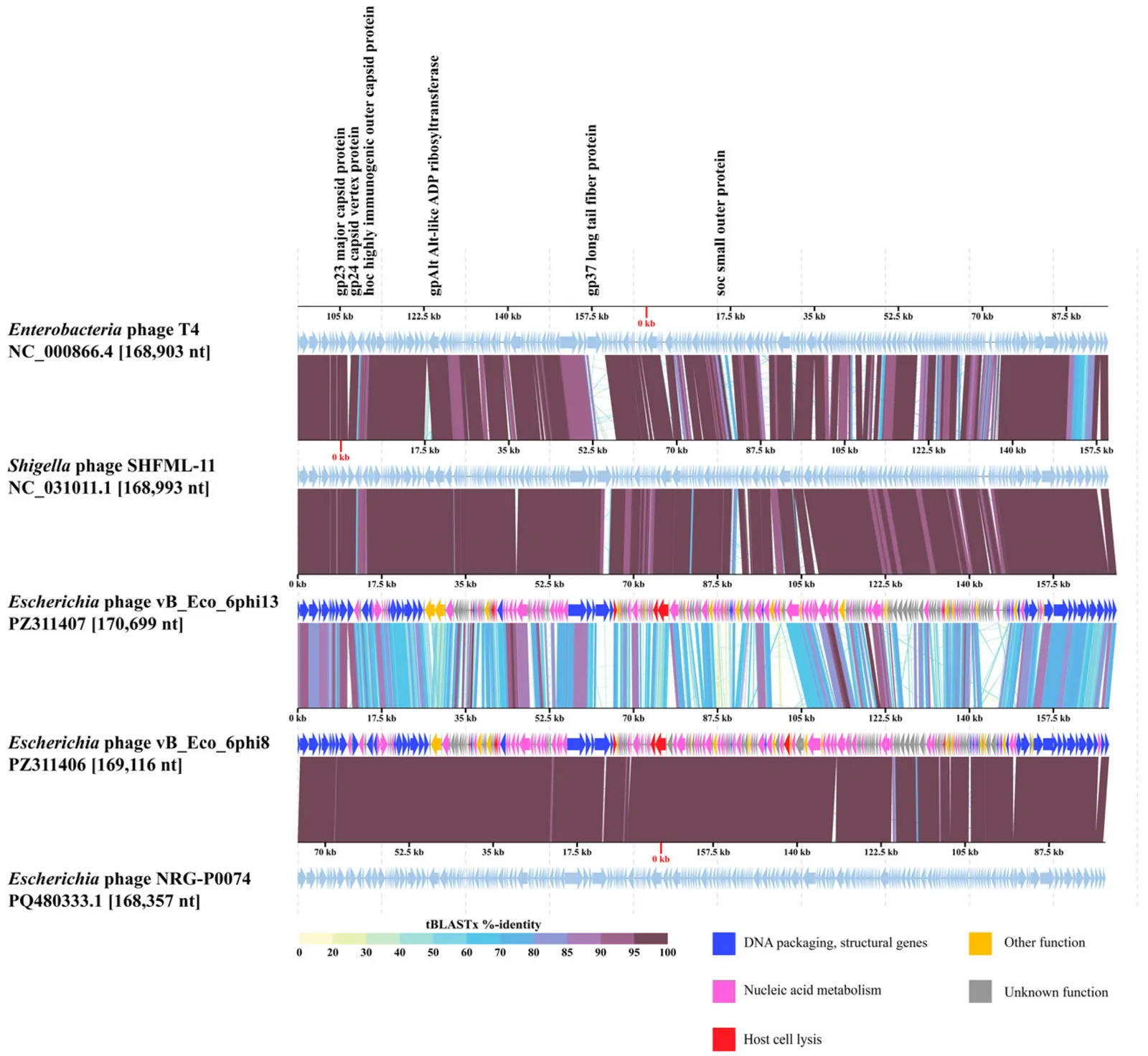
Pairwise comparison of the complete genomes of bacteriophages vB_Eco_6phi8 and vB_Eco_6phi13 with genomes of related phages performed using tBLASTx.

Figure 13 shows a linear alignment (tBLASTx) of complete genomes demonstrating the degree of pairwise similarity between bacteriophage 6phi13 and its closest relatives (including *Shigella* phage SHFML-11 and *Escherichia* phage NRG-P0074), as well as bacteriophages T4 and 6phi8.

## 4. Discussion

This study presents the physicochemical and genomic characteristics of three bacteriophages—6phi8, 6phi10, and 6phi13—isolated from the commercial therapeutic preparation “Sextaphag^®^” (Microgen).

Bacteriophages 6phi8 and 6phi13 belong to T4-like myoviruses, which are characterized by a long contractile tail. In contrast, bacteriophage 6phi10 is a member of T3/T7-like phages and belongs to podoviruses with a short non-contractile tail. All three studied phages form clear plaques, which is typical of obligately lytic bacteriophages incapable of entering a lysogenic cycle. This is supported by the absence of genes characteristic of temperate bacteriophages, including integrases, excisionases, and Cro- and CI-like proteins (Figure 5, Figure 7 and Figure 8). Differences in plaque size may be associated with features of the phage infection cycle, including adsorption efficiency, latent period duration, burst size, and the rate of lysis of infected cells. Additional effects arise from virion diffusion in soft agar, which depends on phage particle morphology and experimental conditions [16,17].

Among the studied phages, 6phi8 exhibited the broadest thermal stability (Figure 2a), whereas 6phi13 was more sensitive to elevated temperature (Figure 2c). Both T4-like phages remained infective over a broad pH range, although only 6phi8 retained partial viability at pH 2.2 (Figure 3a,c). This comparatively high resistance may reflect differences in virion architecture and capsid stabilization.

The physicochemical stability profiles of phages 6phi8 and 6phi13 are generally consistent with those reported for related T4-like bacteriophages. Within the genus *Mosigvirus*, phage vB_EcoM_PhAPEC2 (NC_024794.1; 86.96% nucleotide identity with 6phi8) shows a significant reduction in titer after incubation at 42 °C for 3 days [18], whereas vB_EcoM-RPN187 (OL770074.2; 89.26% identity) remains viable at 4–70 °C after 1 h of incubation, although its titer decreases substantially at 60–70 °C; it is also stable at pH 3.0–10.0 but is inactivated at extreme pH values [19]. Similarly, the *Tequatrovirus* phage vB_EcoM_JB75 (MH355584.1; 90.38% nucleotide identity with 6phi13) retains activity at 4–42 °C, is inactivated at 60 °C, and remains stable over a broad pH range of 3.0–11.0 [20]. These observations are also consistent with data for bacteriophage T4, which retains lytic activity at pH 4.0–10.0 but is destabilized under extreme pH conditions [21]. In contrast to its relatively broad pH tolerance, T4 is more sensitive to elevated temperatures, with thermal inactivation associated with capsid destabilization and release of genomic DNA [22].

The stability of T4-like bacteriophages over a broad range of temperature and pH conditions is determined by the combined contribution of capsid proteins, including the major capsid protein gp23, the head vertex protein gp24, and the outer capsid proteins Soc and Hoc [23,24,25]. According to multiple sequence alignment performed using Clustal Omega (v1.2.4), a high degree of conservation was observed between gp23 and gp24 of bacteriophage T4 and their homologs in phages 6phi8 and 6phi13. The sequence identity of T4 gp23 with its homologs in 6phi13 and 6phi8 was 96.35% and 95.01%, respectively, while for gp24 these values reached 99.3% and 94.6%, indicating strong conservation of these proteins within this taxon. In contrast, the Soc and Hoc proteins exhibited more variable levels of amino acid sequence similarity. The identity of T4 Soc with its homologs in 6phi13 and 6phi8 was 96.25% and 45.33%, respectively, while the corresponding values for Hoc were 91.22% and 59.25%. These differences may be associated with variations in the stabilizing properties of the outer capsid proteins and, consequently, may underlie the observed differences in pH and thermal stability among the studied phages.

The reduction in bacteriophage 6phi10 titer at 50 °C, its sharp decline at 60 °C (Figure 2b), and its activity within a relatively narrow pH range of 6.0–9.0 (Figure 3b) indicate increased thermal and pH sensitivity. Similar characteristics have been reported for T7-like bacteriophages of the family *Autotranscriptaviridae*: heating at 60–70 °C can cause capsid destabilization and release of genomic DNA, whereas optimal stability is generally maintained near neutral pH [21,26]. Closely related representatives of the genus *Berlinvirus* show comparable profiles. Phage vB_CECAV_058 (PQ356389.1; 85.15% nucleotide identity with 6phi10) remains infective at temperatures up to 60 °C and within a pH range of 4.0–10.0 [27], whereas vB_STy-RN5i1 (NC_071037.1; 77.48% identity) is stable at pH 7.0–9.0 and 4–30 °C, shows reduced activity at 45 °C, and is completely inactivated after 1 h at 60 °C [28,29].

Killing assay experiments demonstrated pronounced bactericidal activity of all three phages against *E. coli* MG1655, with the inhibitory effect consistently increasing with increasing MOI (Figure 4). Effective suppression of bacterial growth at low MOI values (0.01 and 0.1) is associated with high productivity of the infection cycle and rapid phage amplification, particularly in the case of phage 6phi10. The observed dynamics, characterized by multiple peaks in optical density at low MOI values for phages 6phi8 and 6phi13, are likely caused by successive rounds of infection, in which newly produced virions initiate repeated infection of the remaining bacterial cells [17]. An additional contribution to this wave-like kinetic pattern may arise from the phenomenon of lysis inhibition, characteristic of T4-like phages, in which repeated adsorption of phage particles onto already infected cells results in prolongation of the latent period and delayed cell lysis. Collectively, this leads to alternating phases of bacterial population growth and subsequent lysis [30,31].

Genome analysis allowed determination of the taxonomic placement of all three phages and revealed several biologically relevant features. Bacteriophages 6phi8 and 6phi13 are characterized by large genomes (~169 and ~171 kbp, respectively), high coding density (~94–95%), and a modular genome architecture typical of T4-like phages. Bacteriophage 6phi8 can be assigned to the genus *Mosigvirus* (family *Straboviridae*), while its nucleotide identity with the closest known phage NRG-P0074 (PQ480333.1) exceeds the ICTV species demarcation threshold (95%), indicating that these phages represent distinct strains within the same species. Bacteriophage 6phi13 shows more than 95% nucleotide identity with Shigella phage SHFML-11 (NC_030953.1), which also suggests their assignment to the same species (genus *Tequatrovirus*, family *Straboviridae*). It is noteworthy that the closely related phages SHFML-11 (NC_030953.1) and SHFML-26 (NC_031011.1) are components of the phage preparation ShigaShield™, used against bacteria of the genus *Shigella*, highlighting the therapeutic potential of this group of phages [32].

The genomes of phages 6phi8 and 6phi13 show a high degree of similarity both in gene block synteny and modular genome organization, as well as in the sequences of encoded proteins (Figure 13). The only exception is the long tail fiber protein (homologous to gp37 of phage T4), which functions in recognition of bacterial surface receptors and mediates the initial adsorption of the phage, thereby determining its host range [33]. The observed amino acid divergence in gp37 suggests differences in receptor specificity between the phages and reflects their adaptation to distinct bacterial receptors.

In the genome of bacteriophage 6phi13 and its closest phylogenetic relative SHFML-11, two paralogous Alt-like proteins with ADP-ribosyltransferase activity were identified (YEO24877.1, YEO24878.1), sharing 43.29% amino acid identity. Enzymes of this class are injected into the host cell together with phage DNA and immediately catalyze ADP-ribosylation of RNA polymerase and other cellular targets, thereby promoting preferential transcription of phage genes from early promoters [34]. The moderate level of identity between the paralogs suggests functional divergence following gene duplication, which is likely associated with an expanded range of ADP-ribosylation targets and enhanced suppression of host defense systems.

The proteome of bacteriophage 6phi8 shows a high degree of identity with its closest phylogenetic relative NRG-P0074 (PQ480333.1), with the exception of several proteins exhibiting moderate divergence, including the single-stranded DNA-binding protein (SSB), an anti-restriction endonuclease, a block of hypothetical proteins of unknown function, and insertions of homing endonucleases. When tested on the ECOR collection, 15.28% of strains were permissive for NRG-P0074 infection; however, a subset of isolates supported adsorption of the phage but did not allow productive infection [35]. The presence of homing endonuclease genes in the genomes of 6phi8 and 6phi13 is a characteristic feature of T4-like phages. Their abundance indicates a high degree of genomic plasticity and mosaic genome organization. These mobile elements are capable of self-propagation via a homing mechanism and contribute to intra- and interspecies genetic exchange and phage evolutionary adaptation [36].

Bacteriophage 6phi10 differs substantially from the other two phages in genome size (40,100 bp), GC content (48.75%), and taxonomic position. Its nucleotide identity with the closest known phages (FE44, BSP161, BA14: 88.44–91.25%) does not reach the ICTV species demarcation threshold (95%), which allows 6phi10 to be considered a representative of a new species within the genus *Berlinvirus* (family *Autotranscriptaviridae*).

Key genomic differences between bacteriophage 6phi10 and its closest phylogenetic relatives (phages BA14 and BSP161) involve both structural genes (tail tube protein A, head assembly protein, and internal virion protein B) and genes associated with nucleic acid metabolism, including serine/threonine kinase, DNA ligase, nucleotidyl kinase, and an H-NS inhibitor, as well as the presence of endonuclease insertions (Figure 11). Previous cross-transcription experiments with BA14 demonstrated that the RNA polymerase/promoter system of this phage group differs functionally from those of T7 and T3 phages [37]. Nevertheless, the high conservation of RNA polymerases among 6phi10, BA14, and BSP161 suggests similar transcriptional mechanisms within the genus *Berlinvirus*.

The identification of a putative novel *Berlinvirus* species expands the known genomic diversity of this genus and provides an additional reference point for comparative analyses of T7-like phages. The divergence observed in structural and nucleotide metabolism genes may reflect lineage-specific adaptations affecting host recognition, intracellular replication, or interactions with bacterial defense systems. However, the functional consequences of these genomic differences require experimental validation.

The predicted DTRs of phage 6phi10 are approximately 200 bp in length, consistent with those reported for T3, T7, and closely related phages, including FE44 (NC_022744.1), ETEP21B (OR979722.1), BA14 (NC_011040.1), Berlin (NC_008694.1), Yepe2 (NC_011038.1), Yep-phi (NC_023715.1), and Kvp1 (NC_011534.1). In T7-like phages, head-to-tail replication concatemers are processed during DNA packaging to generate mature linear genomes containing short exact terminal repeats. Thus, the proposed terminal organization of 6phi10 is consistent with the genome maturation and packaging strategy characteristic of T7-like bacteriophages [38].

Although the studied phages were isolated using *Escherichia coli* MG1655, they are phylogenetically closely related to phages infecting different members of the family *Enterobacteriaceae*, including the genera *Escherichia*, *Shigella*, *Yersinia*, as well as bacteria of the genus *Erwinia* (family *Erwiniaceae*). This reflects the evolutionary relatedness of these bacterial taxa and is consistent with reports of polyvalent bacteriophages capable of infecting bacteria across different genera, including members of the family *Enterobacteriaceae* [39,40,41]. However, based solely on genomic evidence, the studied phages can only be hypothesized to possess a broad host range, which requires direct experimental confirmation.

The taxonomic diversity of the isolated phage strains, belonging to different families (*Straboviridae* and *Autotranscriptaviridae*) and genera (*Mosigvirus*, *Tequatrovirus*, and *Berlinvirus*), may contribute to enhanced efficacy of bacteriophage formulations. The use of phage combinations with distinct infection mechanisms and receptor specificities enables broader coverage of bacterial strains and reduces the likelihood of resistance development compared to the use of individual phages [42,43]. A broader host range could in principle extend cocktail coverage against *Enterobacteriaceae* pathogens beyond *E. coli*, but may also reduce target specificity, increase potential off-target effects on commensal microbiota, and raise additional regulatory considerations for formulation approval.

Thus, this study provides a physicochemical and genomic characterization of three bacteriophages isolated from the “Sextaphag^®^” preparation. Phages 6phi8, 6phi10, and 6phi13 are virulent viruses with well-characterized genomes lacking virulence determinants and elements associated with the lysogenic cycle, and they also exhibit stability with respect to a range of physicochemical conditions. These findings provide a basis for follow-up studies assessing host range and killing efficiency against clinical and multidrug-resistant *Enterobacteriaceae* isolates, which will be required to more fully establish the therapeutic and formulation potential of these phages.

## Figures and Tables

**Table 1:** Key genomic characteristics of bacteriophages 6phi8, 6phi10, and 6phi13.

Parameter	Phages		
	6phi8	6phi10	6phi13
Genome length (bp)	169,116	40,100	170,699
GC content (%)	37.45	48.75	35.28
Number of ORFs	261	44	270
Coding density (%)	94.8	89.4	94.0

## Data Availability

The original contributions presented in this study are included in the article. Further inquiries can be directed to the corresponding authors.

## Abbreviations

The following abbreviations are used in this manuscript:

AMR	Antimicrobial resistance
bp	Base Pair (s)
GRAM	Global Research on Antimicrobial Resistance
LB	Lysogeny Broth
OD	Optical Density
MOI	Multiplicity of Infection
CDS	Coding Sequence
ORF	Open Reading Frame
DTR	Direct Terminal Repeat
ICTV	International Committee on Taxonomy of Viruses
SSB	Single-Stranded DNA-Binding Protein
ADP	Adenosine Diphosphate
